# Updates to the NASA human system risk management process for space exploration

**DOI:** 10.1038/s41526-023-00305-z

**Published:** 2023-09-07

**Authors:** Erik L. Antonsen, Erin Connell, Wilma Anton, Robert J. Reynolds, Daniel M. Buckland, Mary Van Baalen

**Affiliations:** 1 Center for Space Medicine, Department of Emergency Medicine, Baylor College of Medicine, Houston, TX USA; 2 Leidos Innovations, Houston, TX USA; 3 KBR, Houston, TX USA; 4 Duke University, Durham, NC USA; 5 NASA Johnson Space Center, Houston, TX USA

**Keywords:** Risk factors, Research management, Aerospace engineering

## Abstract

This paper describes updates to NASA’s approach for assessing and mitigating spaceflight-induced risks to human health and performance. This approach continues to evolve to meet dynamically changing risk environments: lunar missions are currently being designed and the ultimate destination will be Mars. Understanding the risks that astronauts will face during a Mars mission will depend on building an evidence base that informs not only how the humans respond to the challenges of the spaceflight environment, but also how systems and vehicles can be designed to support human capabilities and limitations. This publication documents updates to the risk management process used by the Human System Risk Board at NASA and includes changes to the likelihood and consequence matrix used by the board, the design reference mission categories and parameters, and the standardized evaluation of the levels of evidence that the board accepts when setting risk posture. Causal diagramming, using directed acyclic graphs, provides all stakeholders with the current understanding of how each risk proceeds from a spaceflight hazard to a mission-level outcome. This standardized approach enables improved communication among stakeholders and delineates how and where more knowledge can improve perspective of human system risks and which countermeasures can best mitigate these risks.

## Introduction

As the scope of human spaceflight expands, the risk to space travelers expands as well. Together with international partners, the National Aeronautics and Space Administration (NASA) has crewed the International Space Station (ISS) for the last 23 years. After the retirement of the Space Shuttle in 2011, the ISS became NASA’s primary effort of human spaceflight, and systems and approaches were tailored specifically to ISS mission parameters and risks. These ISS missions in low Earth orbit (LEO), which can stretch from 30 days to one year, are one type of design reference mission (DRM) that NASA evaluates for risk to human health and performance. With the flight of the first astronauts on commercial vehicles to the ISS in 2021 and the launch of Artemis I in 2023, plans to return humans to the moon, and a human mission to Mars rapidly approaching, mission types that have historically been considered futuristic are now far into their design and development phases. It is well understood that human spaceflight is a specialized endeavor that is organized and regulated differently than terrestrial environmental and occupational health risks. As astronauts go further from Earth and are subjected to other human spaceflight hazards, those differences increase and require specialized attention.

As NASA conceptualizes and designs the vehicles, suits, systems, and missions that will allow humans to travel beyond the ISS, the processes for addressing and communicating risk posture and mitigation needs must also evolve^1^. New challenges will be encountered because new vehicle designs and mission tasks will place unprecedented demands on astronauts’ capabilities during a mission^2,3^, and health risks are expected to escalate with lengthening duration of exposure to the spaceflight environment and distance from Earth^4–7^. Those same extended spaceflights are expected to increase risk to the long-term health (LTH) of astronauts after the mission^8^. Finally, the Artemis missions, which will return astronauts to the Moon, consists of an integrated set of flight programs that have separate program managers and teams. In this context, a “program” describes a formally funded NASA spaceflight program such as the Space Shuttle Program, the ISS Program, or the Commercial Crew Program. In Artemis, the Orion Program’s vehicle will carry astronauts to the lunar vicinity to dock with the Gateway Program’s lunar orbital space station, and descent to the lunar surface is under the purview of the Human Landing System Program. Integrating these 3 flight programs adds to the already complex crew-vehicle design challenges of any one of those vehicles and increases the level of mission complexity as well. An interdisciplinary approach will be required to integrate these flight programs and will result in complex systems that weigh the health, performance, and medical needs of astronauts against the engineering realities of the vehicle, habitat, and spacesuits^9–13^.

Romero and Francisco^14^ published the last public update to the process for managing spaceflight-induced risk to humans (hereafter referred to as human system risk), which was used by the Human System Risk Board (HSRB) at NASA Johnson Space Center until 2018. Subsequent changes to this process were generated in response to changes in human spaceflight needs, and a new Human System Risk Management Plan (RMP) JSC 66705 Revision A that documented the changes, was released internally at NASA in October, 2020^15^. The HSRB uses the processes described here to provide transparent guidance during discussions of prioritization that explicitly state the priorities of the Health and Medical Technical Authority (HMTA) as a non-advocate. The goal of these processes is to systematically reduce total risk to astronaut crews. The HSRB piloted several updates to these processes that were evaluated to determine if they could be implemented by the board, including.

- updates to definitions of key terms to improve alignment and communication among stakeholders.
- formal definitions of risk drivers and a risk mitigation framework.
- updates to the design reference missions (DRM) categories and their parameters used by NASA to align risk discussions with changing mission priorities.
- changes to the process for assessing the levels of evidence used by the HSRB.
- established principles for prioritizing risk characterization and mitigation efforts that were agreed upon by the stakeholder community.
- directed acyclic graphs (DAGs) to improve communication and enable configuration-managed causal diagramming of risks.

The HSRB assesses human system risk by DRM category, which is defined by destination, operating environment, and expected mission duration. DRMs are used to provide continuity of expected high-level mission parameters in lieu of the constantly changing attributes of specific mission proposals and future undefined missions that are determined at the Mission Directorate level within NASA. Because only a small number of humans have flown in space, significant uncertainty exits regarding how short-term and long-term exposure to the spaceflight environment changes human health and performance. Changes to human health and performance can adversely impact an astronaut’s ability to perform critical tasks tied to mission objectives and can affect their ability to be recertified for flight status after their spaceflight mission. Human system risks also address the long term health (LTH) effects of exposure to the spaceflight environment, effects that extend beyond the end of a flight program. The HSRB must ensure that the knowledge gained through human spaceflight and complementary advances in applicable terrestrial medicine are captured, documented, and applied to reduce the risks crewmembers will face during current spaceflights and future exploration missions. To accomplish this, a formal continuous risk management process is used to ensure that new evidence gleaned from flight operations and research effectively feeds back into risk assessment. This information is intended to help NASA make risk-informed decisions that protect the astronauts and the mission. The details of that process, described below, include updates to the formal definitions and processes that the HSRB uses to track risk posture for the 30 human system risks and concerns (as of July 2023) that are currently being managed by the board.

## Risk management process update

### Key definitions updates

The HSRB has a variety of stakeholders who include experts in many fields such as spaceflight operations, engineering, medical, life sciences, performance, human factors and human system integration (HSI), and more. At times, these experts can have different interpretations of commonly used terminology at NASA. To address this, Rev. A of the RMP included formal definitions to ensure that the use of specific terms carry a common meaning among different experts.

A human system risk is a recognized potential undesired flight crew health or performance outcome that has a clear consequence and attendant likelihood (likelihood and consequence [LxC]) supported by evidence for a given DRM category.

A human system concern is a potential undesired human health or performance outcome for the crew for which there is insufficient evidence to allow an LxC assessment for any DRM.

The risk posture is an agreed upon understanding of the state of a human system risk that is based on the best available evidence. This is decided by the HSRB based on assigned DRM-specific LxC scores and their drivers and underlying assumptions. Risk posture is communicated through associated risk scores, colors, dispositions, and rationales. The HSRB uses risk posture to communicate human system risk for a given mission.

Risk disposition represents the HSRB’s official position on the current state of the risk for a given DRM that assumes known countermeasures and monitoring will be implemented. Eight options exist for HSRB risk disposition: requires characterization, requires mitigation, requires mitigation/standards refinement, accepted, accepted with monitoring, accepted with optimization, transferred, and retired. A risk is accepted by the board when countermeasures are deemed effective and efficient or no further risk reduction is considered appropriate at that time. These dispositions are fully described in the RMP^15^.

Spaceflight hazards—In their 2020 report, Romero and Francisco^14^ reviewed the 5 spaceflight hazards listed below, which guide the derivation of risks: Altered gravity, Radiation, Isolation and confinement, Hostile closed environment, and Distance from Earth.

These hazards are the evolving aspects of the spaceflight environment that are harmful to humans. They are understood to be the fundamental causes of spaceflight-induced risks to humans in the sense that they induce new challenges from the moment a human is launched into space. “New” here refers to a comparison with challenges faced by humans on Earth. For the purposes of risk management, it is not sufficient to simply list and describe these spaceflight hazards; we must also understand their potential impact to astronauts and mission-level outcomes.

The challenge of aligning perception of the terms used by various sets of experts within NASA also extends to the risks themselves. Different experts carry different mental models of what a specific human system risk is, what factors contribute to the development of that risk, and the importance that a given risk should carry when prioritizing research investments or operational capabilities within the constraints of budget and flight capacity. Therefore, the processes, DRMs, risk assessments, and other RMP aspects have been refined to improved clarity.

#### The continuous risk management process

**Fig. 1:**
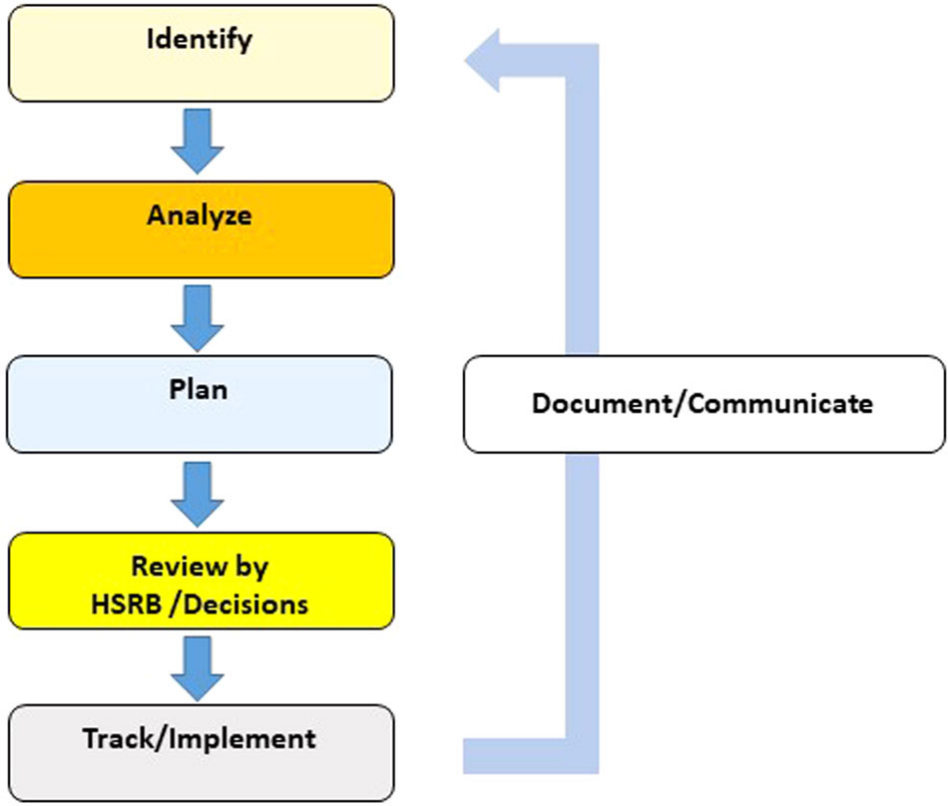
The continuous risk management process for human system risk management at NASA^15^. HSRB Human systems risk board.

The HSRB implements formal processes to track and manage human system risks. An overview of the risk management process using continuous risk management principles is shown in Fig. 1.

This process was described by Romero and Francisco and has not significantly changed with this update in risk management process^14^. The HSRB reviews each human system risk every 1–2 years and formalizes this process through configuration managed steps that include identifying, analyzing, planning, deciding, tracking, and implementing risk products within a continuous process of documentation and communication. Although the high-level continuous risk management process has not changed, many of the details and guidance that the HSRB provides have been updated, which are summarized here along with rationale and context.

The identify phase has 2 parts: to identify if there are any new risks or concerns that should be formulated and tracked; and to identify new evidence that may influence understanding of the risk posture that warrants further analysis. Once new evidence has been collected for a risk update at the HSRB, the analyze phase begins. Once the analyze phase is complete, the information must be used to drive decisions on risk mitigation. This is performed in the plan, review, track, and communicate steps. Analyze includes 7 steps.

- Identifying risk drivers
- Understanding risk DRM applicability
- Delineating relevant risk impact categories
- Assigning LxC scores
- Assigning risk dispositions and rationale
- Communicating supporting level of evidence (LoE)
- Summarizing risk posture information

The steps that have relevant updates are explained below.

##### Risk drivers

The risk drivers were defined and included in the RMP updates to ensure that stakeholders understand how risk can change with different mission parameters. Early in the systems engineering processes for mission design, requirements are generated that ultimately define the level of risk that will be encountered in a mission^10,16^. If mission attributes change later in the design process and requirements are not revisited, the initial assessment of risk may no longer be a valid representation of the risk expected during a given mission.

Risk drivers describe how the spaceflight hazards modify risk posture depending on variation in mission attributes. Risk drivers are not risk-specific, they change depending on the mission objectives and can increase multiple human system risks. Identifying the potential drivers of human system risks allows (1) a clearer understanding of the origin of the risk and potential areas for risk mitigation, (2) an improved understanding of the potential relationships between risks, and (3) an improved ability to prioritize risks for stakeholders. Table 1 lists the risk drivers pertinent to human system risks; these are taken from the Human System RMP Revision A^15^.

**Table 1:** Mission attributes that drive risk to the human system and examples of how the risk drivers affect risk from specific mission parameter.

**Time: Exposure to spaceflight hazards increases with mission duration**^14^	
**Gravity Environment**	Exposure to a gravity environment that is less than Earth-normal begins a process of adaptation; some of these adaptations create issues because human bodies have evolved to function in a 1 G environment. Increasing duration of exposure leads to increasing deconditioning.
**Radiation Environment**	Risk from exposure to space radiation is both duration-dependent and intensity dependent and may have in-mission or long-term health impacts.
**Isolation and Confinement**	As the period of isolation during a space mission increases, the risk of psychological, physical, and mental health issues increases.
**Hostile Closed Environment**	Perturbations in vehicle or spacesuit conditions (air quality, temperature, accelerations, movement restriction, etc.) can result in illness, injury, or inability to perform critical tasks, and risk increases over time of exposure. Examples include launch and landing loads, CO_2_ levels in the spacesuit and vehicle, and amount of time spent in a hot or cold environment due to insufficient capability of the environmental control and life support system.
**Distance from Earth: Distance from Earth affects the energy and cost associated with mass delivery as well as communications and logistical factors**	
**Communications Delay**	As distance from Earth increases, communication lags will delay ground support to crews and operations will shift from real-time support to greater crew autonomy, implementation of intelligent support software, and store-and-forward communications^22,23^.
**Time to Definitive Care (Evacuation Time)**	As distance from Earth increases, the time required to deliver medical care increases. Medical evacuation timeframes must be considered as drivers of health risk for crews. In particular, for Mars DRMs, medical evacuation will not be possible and this shifts the risk posture for crews^3,4^.
**Consumables Resupply**	As distance from Earth increases, design and operational system trades are likely to target the mass and volume needed for food, pharmaceuticals, medical equipment, and consumables. If resupply is possible, the risk of interruption of the supply chain becomes greater with greater distance from Earth. For Mars DRMs, no resupply options will be available and pre-supply options have severe disadvantages due to shelf life^5,7^.
**Vehicle Resource Constraints**: The limitations on mass, power, and volume will be determined by the mission goals and attributes. Different mission types will carry different risk postures based on the total available mass, power, volume, and data bandwidth that can be traded among vehicle systems.	
**Vehicle Habitable Volume and Capability**	The levels of risk from hazards such as isolation and confinement and closed/toxic environments is heavily dependent on net habitable volume, which is different from total resource volume. Limited habitable volume may result in the restriction or exclusion of private crew quarters and amenities that can help offset behavioral and interpersonal issues. Decrements in individual and team performance are expected as capabilities and countermeasures are sacrificed.
**Crew Selection and Assignment**	Medical and behavioral profiles of crewmembers must be understood, formalized into standards, and accommodated in mission planning. If crews are composed of a mix of government and private crews, this may result in more medical and behavioral risk because it is unclear if commercial and private astronauts will undergo the same selection procedures (both medical and psychological screening) and the same teaming evaluations as NASA astronauts and other government-sponsored astronauts^24–27^.
**High Risk Activities: Certain missions will require tasks and activities that pose greater risk to both crew and mission**	
**Extravehicular Activities (EVAs)**	Increasing the number of EVAs increases the likelihood of decompression sickness and suit- or activity—related injuries^28–31^. If crews cannot shelter effectively during a solar particle event during an EVA, this may affect the likelihood of acute radiation sickness^32,33^.
**Beyond Low Earth Orbit**	Travel outside Earth’s magnetic sphere increases the radiation exposure of crews beyond that which has been experienced in low Earth orbit^33,34^. In the case of EVA activity this can result in increased damage from solar particle events and may limit mission activity.
**Orbital Mechanics:** Orbital mechanics may extend the time required to return a sick or injured crewmember to the Earth for definitive care, so NASA may be forced to prioritize between mission objectives and loss of crew life/permanent disability to astronauts for some types of missions. A realistic assessment of the probability of a medical condition occurring, the complexity of resources needed to treat these conditions, and the potential futility of treatment for some severe medical conditions should drive prioritization that ensures a reasonable match between medical need and medical capability^35^.	

##### Design reference missions (DRMs)

Recognition and articulation of risk drivers are intended to give stakeholders insight into the attributes of the DRMs being assessed. As NASA’s mission interests change, programs may modify significant portions of their concepts of operations, which in turn affects the level of human system risk for a mission. Table 2 shows the updated set of DRMs used by the HSRB, including associated assumptions relevant to risk assessment. The factors considered are derived from the risk drivers above and are presented in a fashion conducive to quantitative analysis.

**Table 2:** Updated design reference missions currently in use by the Human System Risk Board.

DRM Categories	Mission Type and Duration	Gravity Environment	Radiation Environment	Vehicle/Habitat Design	Distance from Earth		EVA Frequency
					Evacuation	Communication	
**Low Earth Orbit**	Short ( < 30 days)	Microgravity	LEO – Van Allen ( < 5–15 mGy)	Mid-sized volume, resupply	1 day or less	Real-time	1–4 EVAs
	Long (30 days–1 year)	Microgravity	LEO – Van Allen (5–150 mGy)	Mid to large optimized volume, resupply	1 day or less	Real-time	1–10 EVAs
**Lunar Orbital**	Short ( < 30 days)	Microgravity	Deep Space – Van Allen (15–20 mGy)	Small volume, self-contained, resupply	3–11 days	Real-time	Contingency EVA only or very few EVAs
	Long (30 days–1 year)	Microgravity	Deep Space (175–220 mGy)	Mid-sized volume, self-contained, limited resupply	3–11 days	Real-time	Contingency EVA only or very few EVAs
**Lunar Orbital + Surface**	Short ( < 30 days)	Microgravity & 1/6 G	Deep Space – Van Allen (15–20 mGy)	Small volume, resupply	3–11 days	Real-time	5 EVAs, some back-to-back
	Long (30 days–1 year)	Microgravity & 1/6 G	Deep Space (100–120 mGy)	Mid-large sized optimized volume, limited resupply	3–11 days	Real-time	3-4 EVAs per week, 20–24 EVA hours per week
**Mars**	Preparatory ( < 1 year)	Microgravity	Deep Space (175–220 mGy)	Mid-sized optimal volume, limited resupply, closed loop environment	Days-weeks	Controlled - Delayed	Contingency EVA only or very few EVAs
	Mars Planetary (730–1224 days)	Microgravity & 3/8 G	Deep Space - Planetary (300–450 mGy)	Mid-sized optimal volume, no resupply, closed loop environment	Mission duration	No real-time	2 crew x 8 h EVA x 20 EVA days

*EVA* Extravehicular activity; *LEO* Low Earth orbit.

These DRMs are broken into the 4 primary mission types that are relevant to current or anticipated programs. The LEO DRM includes short-duration missions similar to commercial spaceflight missions, and long-duration missions similar to ISS missions. The lunar orbital DRM includes short- and long-duration missions that apply to Orion and Gateway. The lunar orbital + surface DRM include both Orion and Gateway with the addition of the human lander system and lunar surface extravehicular activities (EVAs). The Mars DRM includes preparatory and planetary missions to account for the simulation and testing of new technologies that are likely needed prior to the full-scale Mars mission. Because the preparatory DRM is an analog of the Mars mission, it is considered to have different mission attributes than the actual planetary Mars missions.

##### Risk impact categories

**Fig. 2:**
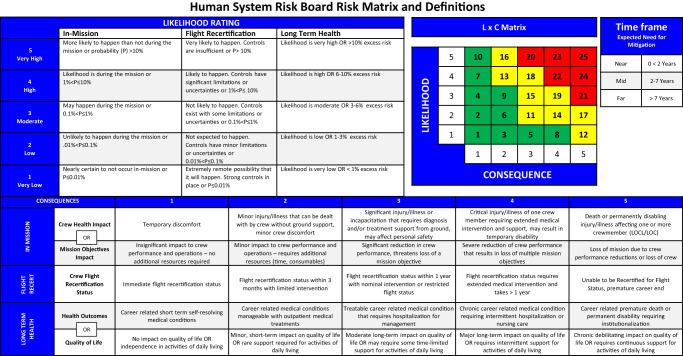
Human system risk board risk matrix and definitions. LXC Likelihood and consequence.

Because risk consequence can impact the crew and/or NASA mission objectives, risk impact categories were created to enable construction of a risk matrix that reflects both possibilities. The 3 risk impact categories are used in the LxC matrix in Fig. 2. As defined, a risk may be applicable to multiple categories.

In-Mission Risk—the risk posture for crews during a mission is defined from successful launch until successful and safe egress from the landing vehicle. The *crew health* impact subcategory identifies health issues, and the *mission objectives* impact subcategory identifies crew task performance issues that may result in loss of mission objectives if realized.

Flight Recertification—in some cases, exposure to the spaceflight environment affects the crewmember’s physical or mental health after a mission, delaying their flight certification and flight recertification status. This applies throughout the career of an astronaut. Although this is not often used in practice, it is included because this risk not only affects the crew, it also affects NASA’s available pool of veteran astronauts who qualify for different flight programs.

Long Term Health (LTH)—is the lifelong effect of spaceflight on physical and mental health and performance of astronauts. The LTH category now consists of the *health outcomes* impact subcategory, which includes medical conditions resulting from career exposures to the spaceflight environment, and the *quality-of-life* impact subcategory, which identifies decrements in the ability of an astronaut to perform daily living activities after a mission because of career exposure to the spaceflight environment.

##### Likelihood x Consequence (LxC) scoring and colors

Central to the risk assessment is determining the LxC score as applied to each DRM and to the different risk impact categories. Each LxC score is assessed by considering the level of supporting evidence and is assigned a color in the risk matrix. Accompanying an LxC score is a risk disposition that defines the HSRB’s overall position on the state of the risk assuming known countermeasures and monitoring that will be implemented in each DRM. Each risk has a summary table that includes these parameters, and the HSRB uses a risk roll-up chart to communicate a comparative assessment of all the risks. The HSRB updated the risk matrix from the 3 × 4 matrix shown in Romero and Francisco^14^ to a 5 × 5 matrix that is based on the risk matrices more commonly used by NASA programs^15^. The 5 × 5 matrix and scale definitions, shown in Fig. 2, adds granularity to risk assessments and helps improve communication with spaceflight programs.

The determination of the LxC scores is based on the following approach using the best available evidence applicable to the particular DRM being assessed: For each risk and DRM, the most probable consequence within the applicable risk impact categories described above is scored from 1 to 5 based on the definitions provided. The associated likelihood for the consequences is then scored from 1 to 3 based on both qualitative and quantitative definitions provided. The choice of the most probable consequence in the assessment helps focus the risk on more reasonable scenarios than extremely low likelihood worst case scenarios. The assigned scores consider uncertainty based on the state of the evidence evaluated. Each risk will be assessed at least 8 LxC scores based on 4 DRM categories broken down into 2 mission types (short and long) for at least 1 risk impact category (up to 3). These scores are plotted in the 5 × 5 grid of likelihood and consequence (in Fig. 2) and will have an associated color and number. The evidence assessment and the resulting LxC scores are reviewed by the HSRB along with the other risk information to support risk posture determination (discussed below). The number in the LxC grid, which is called the risk prioritization score, is discussed further in the section on risk prioritization principles below. Next to the grid is a timeframe box that shows 3 categories for the expected need timeframe for mitigation.

##### Levels of evidence definition and assessment

Underpinning any assessment of risk posture or assertions about risk is the supporting evidence. Spaceflight causes changes to the human body that become a source of risk, and the duration of the spaceflight drives the magnitude of that risk. The Level of Evidence (LoE) scoring process was revised in the current process for managing the human system risk because the prior process required clarification and improvement. The new process includes explicit standards for determining the quality of evidence considered, modifying the LoE scale to move from correlative language specific to epidemiology to causative criteria that is broadly applicable to the broader sources of evidence considered by the HSRB, and clarifying the value of various types of data and evidence when attempting to draw conclusions that are relevant to the human system in spaceflight. The evaluation of the evidence base results in an LoE score assigned alongside each assessed LxC score. These are documented and discussed in more detail in the RMP and in another publication^15,17^.

##### Summarization of risk posture

Table 3 shows an example of a summary of the risk information for a given DRM: in this case a LEO DRM and the risk to crew health due to electrical shock. The DRM category is identified in the far-left column, the next column shows the mission duration (short and long). The LxC information for the in-mission operations and LTH risk impact categories include information on the currently understood likelihood case and related consequence case. The most appropriate LxC option is chosen by the risk custodian team and approved by the HSRB. For both in-mission and LTH categories, risk dispositions, risk disposition rationales, and LoE score represent the risk posture. In this table the green color indicates low risk whereas yellow or red would indicate mid or high-level risk. A table like this is created for each DRM and used by the HSRB as a high-level communication and reference tool.

**Table 3:**
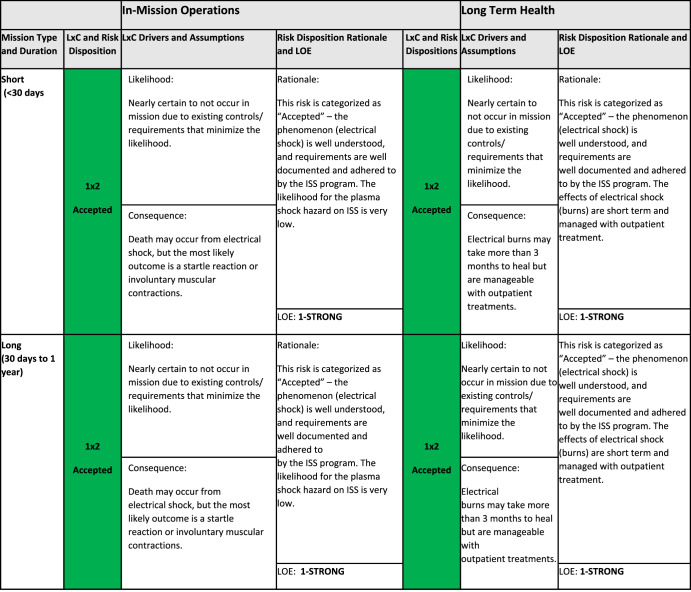
Summary of risk posture for the risk to crew health due to electrical shock (January 2021) for the low Earth orbit (LEO) design reference mission (DRM).

*LxC* Likelihood and consequence, *LOE* Level of Evidence. Green color indicates the risk level as calculated using the risk matrix in Fig. 2.

A risk roll-up table is created to provide insight into the full complement of risks. Table 4 shows the roll-up table for the 30 human system risks that is current as of July 2023. A high-level overview of all the risks is presented across all the DRMs under consideration. The color assignments are a function of the risk matrix shown in Fig. 2. It is important to note that the colors are not an indication of whether a risk should be used to stop a mission from occurring. Instead, they are intended to convey only relative risk levels to help identify opportunities for investment of resources or to justify recommendations to decision-makers. Maintenance of this table also tracks reduction in risk over time.

**Table 4:**
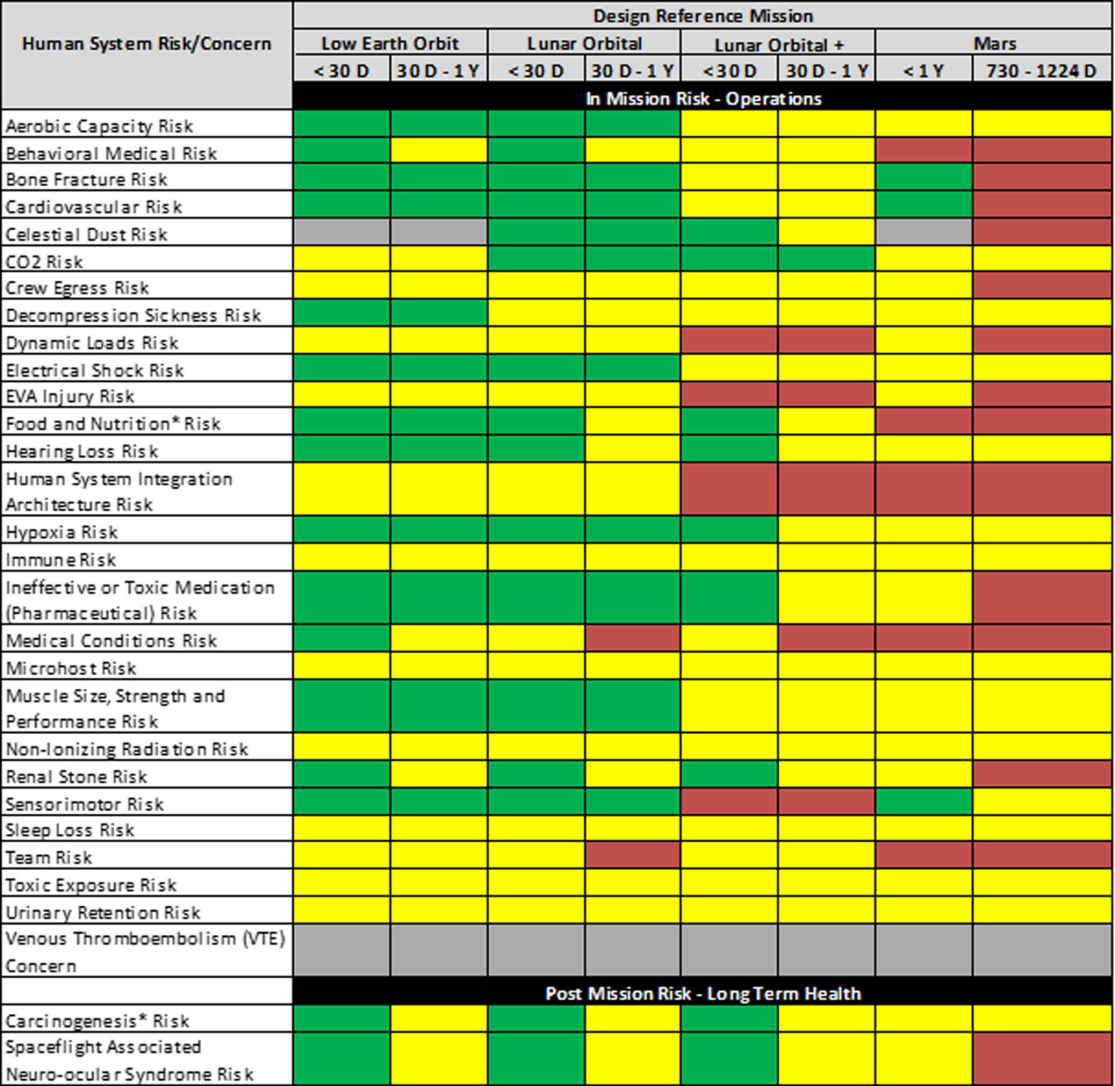
Summary of all the human system risks mapped across the set of design reference missions.

Current as of July 2023. The assigned color-coded risk postures red, yellow, and green denote high, mid, and low risk postures, respectively. These are determined using the risk matrix in Fig. 2. *D* Days, *Y* Years, *Pharm* Pharmaceutical.

#### Risk mitigation framework

Once risk has been assessed and scored, the question of how risk is mitigated becomes relevant. The framework for risk mitigation is designed to compel program and project managers to identify how a particular investment is expected to help mitigate human system risks. The 5 categories are used to help clarify expected benefit of risk mitigation activities. Deliverables such as scientific research, occupational or clinical surveillance measures, standards development, technology investments, flight rules, etc., must contribute to one of the following categories to be considered by the HSRB as useful for risk mitigation.

- *Risk Characterization*—Deliverables in this category contribute to understanding the nature of the risk—how and why the risk occurs—and enables plans to decrease likelihood or consequence based on that understanding. Characterizing the risk requires an understanding of the magnitude of the impact of that risk on spaceflight crews. This helps identify when a risk is worth investing in and when it should be down-prioritized in favor of other risk investments.
- *Prevention (Hazard Control)*—These deliverables identify ways to prevent risks from occurring or to decrease the likelihood they will occur. Examples include crew selection recommendations, human system integration recommendations, standards recommendations, clinical practice guidelines, and flight rules.
- *Consequence Reduction*—Prevention of all risk is impossible, so countermeasures that intervene or treat a problem are required for human spaceflight, and as the distance from Earth increases, intelligent selection of these countermeasures may be mission enabling. These deliverables identify approaches that will reduce the severity of problems that could have adverse effects on crew health or on mission objectives. For example, countermeasures, healthcare monitoring, diagnosis and treatment resources, and clinical practice guidelines all provide intervention capabilities that reduce the consequence of an event that has occurred.
- *System Resilience (Improving Margin)—*These deliverables identify system improvements that may directly or indirectly improve posture of human system risk by helping to improve crew resilience in accomplishing mission objectives. In this case ‘system’ refers to the vehicles, habitats, space suits and humans and how they are integrated together, which can be thought of as the total system margin to tolerate error or off-nominal operations. This category includes technologies that enable system improvements such as decreased need for valuable mass, power, volume, or data storage or bandwidth requirements. These savings increase the likelihood that risk mitigation technologies will be included within the tight mass and volume restrictions of exploration missions. In some cases, individual risks that are yellow or green may contribute a large or synergistic effect on the system as a whole. In these cases, continued risk mitigation and investment may be warranted to help reduce total system risk.
- *Risk Acceptance—*Deliverables that provide information to support a decision regarding acceptance of a moderate or high risk are included in this category. These may include information on return on investment or cost and schedule limitations, which can initiate discussion about whether investments for the risk in question are best moved to other areas.

Although the first 3 categories are intuitive, the historical approach to risk-related research and data collection has been siloed, in part, by the structure of the risks themselves. The HSRB encourages research investments that target improved system resilience as a means of reducing overall system risk. The design of the crew health and performance system through rigorous human system integration processes is one approach to improving system resilience. Residual system risk is difficult to characterize when research focuses primarily on silos of specific risks alone. Additionally, the work that NASA performs to understand and mitigate risk is applied toward a specific goal*—*achieving acceptance of an appropriate amount of human system risk within the larger context of vehicle and mission risk. The HSRB encourages investments in activities that help determine when risk has been sufficiently reduced. Although eliminating all human system risk is an admirable goal, it would likely result in increases in vehicle or mission risk that would obviate the gains achieved^18^. As such, the HSRB does not advise further investments beyond an appropriate level of risk acceptance, because that would have a poor likelihood of return on investment.

#### High value risk mitigation targets

Using the 5 risk mitigation categories, risk custodian teams identify high value risk mitigation targets and recommend investments and countermeasures that should reduce risk. These typically include areas where major gaps in knowledge or capability exist, or other targets that could yield returns worthy of investments in time, money, and other resources. These recommendations represent the risk custodian’s and the HSRB’s perception of the best near-term targets for reducing risk from a total system perspective.

#### Risk prioritization principles

In the context of research investments, limited budget is available to address the myriad of potential research projects that could improve risk posture. Similarly, the systems engineering lifecycle and design process for vehicles, habitats and spacesuits have limited mass, power, volume, and data-bandwidth to accommodate all the potential countermeasures that can be envisioned to improve crew health and performance. Many subject matter experts (SMEs) have deep insight into specific spaceflight problems, but few have broad insight into the spectrum of needs and the full set of constraints placed on the crew health and performance system, therefore, the HSRB provides a non-advocate approach to help stakeholders prioritize investments and capabilities that are being considered for human spaceflight missions. Non-advocate in this context means that the HSRB is responsible for tempering the enthusiasm brought to any specific risk, project, or capability, and honestly weighing the potential value against the potential cost, which can include budget, schedule, changes in risk posture, or displacement of other important research or capabilities. The HSRB holds this responsibility because it is a health and medical technical authority board, and in this context the HSRB works with the stakeholders to define criteria that can help prioritize decisions. Briefly these include:

- *Risk Prioritization Score*—The severities of risks are communicated at highest level using colors. However, for prioritization, the LxC scores, shown as the numbers in each of squares of the 5 × 5 matrix in Fig. 2, identify the comparative urgency of resolving the risk. This single metric is used to identify “red risks”. For many years these scores were the sole discriminator for assessing priority, however, additional discriminating metrics should be considered.
- *Risk Hierarchy*—The more fundamental risks likely require a certain level of mitigation before other dependent risks can be mitigated. For example, the basic human needs of food and nutrition must be met before mitigating the aerobic performance risk.
- *Risk Dependency*—All the risks are simultaneously present in a mission despite the tendency to silo them for ease of research. Where possible, the risks whose nature or countermeasures are likely to affect many other risks should be prioritized over the risks that have few interconnections with other risks. For example, the design of the vehicle is a part of the human system integration architecture (HSIA) risk. This risk is mitigated, in part, by including experts in human systems integration at every step of the vehicle design, which also ensures that human system countermeasures for other risks are also included. As such, the HSIA risk is linked to the successful mitigation of all the other risks. Directed Acyclic Graphs (DAGs) were constructed to help identify these links between risks, and to show the currently understood causal flow from spaceflight hazards to mission-level outcomes for each risk. Points of known or suspected interconnection between each risk are mapped in the DAGs, which are configuration managed by the HSRB. These DAGs discussed in more detail elsewhere^19^ and are summarized in the next section.
- *Need Timeframe*—The time available to mitigate a risk varies by the specific mission type and specific risk. For example, the risk of radiation-induced carcinogenesis is managed and accepted for both long and short missions in LEO. However, the radiation exposure significantly increases for Mars missions or long-duration lunar missions. A longer lead time will be available to effectively mitigate this risk than the lead time available to effectively mitigate risks from EVA given the earlier calendar dates for lunar surface missions.
- *In-Mission Risk vs. LTH Risk*—Although LTH effects that occur either during or after an astronaut’s career must be mitigated, it is incumbent on NASA to prioritize in-mission risks over LTH risks to crews. Astronauts accepted a 1:90 risk of loss of life at the later phases of the Space Shuttle program, and as high as 1:10 in the early phases of the program^20^. NASA enables human spaceflight in the best achievable risk posture; however, if LTH concerns were to over-ride in-mission concerns, astronauts would never fly.
- *Expected Investment Benefit*—Historically NASA is the leading source of research investment in human needs in spaceflight. Because of this, NASA has focused their investments on developing resources that can mitigate spaceflight risk. Other agencies and funding sources support research that targets human health and performance challenges on Earth. These are often much larger investments than NASA is able to provide. If technology developed by other funding sources is likely to reduce a risk faster or more successfully than NASA specific investments, that risk should receive less priority when considering NASA’s limited funding availability. For example, astronauts are exposed to higher levels of radiation during spaceflight than on Earth and this may induce a greater risk of developing cancer. The National Institutes of Health invests far more in attempting to cure cancer than NASA can or should. Therefore, rather than also funding research into cancer treatments, NASA should prioritize their investments into strategies such as characterizing the unique effects of space radiation or optimizing vehicle shielding.

The HSRB uses the principles described to reduce total risk to astronaut crews. If risk mitigation efforts in the human domain are over-stated, this could displace mass or volume that may be needed for other mission systems, and this could raise total mission risk while appearing to improve the human system risks.

#### Causal diagramming

To provide a metric for quantifying risk dependencies, a pilot program was instituted to construct DAGs for each of the 30 human system risks. This is described more completely elsewhere^15,19,21^, however, it is discussed here for context. One of the biggest challenges when a wide variety of experts discuss the risk reduction process is the lack of a shared mental model of the causes of spaceflight-induced risk. It is a common bias for SMEs to overstate the importance of their own area of expertise while understating the importance of other domains. This is a natural part of attempting in good faith to contribute to the larger systems problems faced by NASA. It is also difficult for non-experts to understand why a particular research project or medical capability may be important. To help address this problem, the DAGs show the causal flow of risk that begins with immersion in the spaceflight environment at launch, and through the many dependent contributing factors that lead to increased likelihood of adverse mission-level outcomes. Each of these DAGs were created using strict criteria and structure to enable like-to-like comparison of the risks and to map the known or suspected interactions between the risks at the level of contributing factors or countermeasures.

In the context of risk, the DAGs depict the relationship between important contributing factors that affect health and performance. ‘Health’ in this case refers to the absence of medical conditions that are likely to harm or cause decrements in performance needed to achieve mission objectives. ‘Performance’ typically refers to the individual crewmember’s ability to successfully complete tasks as assigned over the course of a mission. It is known that health and performance does change during spaceflight, but it is helpful to elucidate how those changes can lead to unsuccessful task performance and possibly loss of mission objectives.

**Fig. 3:**
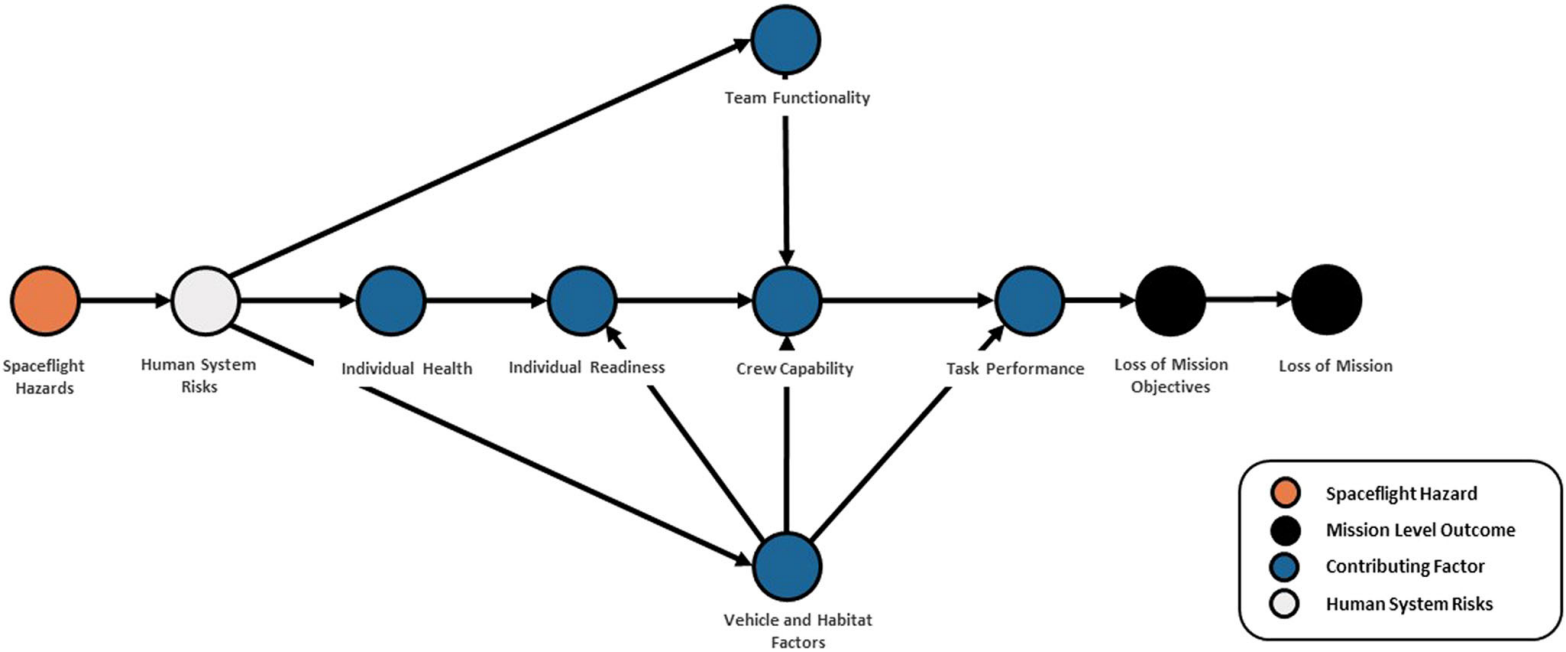
Overview of key causal relationships that affect performance in human spaceflight. This notional-directed acyclic graph shows the progression from the hazards of the spaceflight environment encountered at the time of launch through to mission-level outcomes that include loss of mission objectives and loss of mission.

Figure 3 illustrates the causal chain of performance visualized as a DAG. Task performance is often thought to start with individual readiness, however, in the human system risk domain the causal chain begins with the hazards astronauts are exposed to when they are launched into space. Exposure to those hazards leads to the issues identified as the human system risks, which affect individual health over time during a mission through physiologic changes and deconditioning. During short-duration spaceflight, the effects on an individual may be minor, but for long-duration spaceflight they can lead to incapacitation over time. Health decrements can contribute to decrements in an individual’s performance, but additional factors including the team and the systems involved can affect the performance of mission critical tasks. Individual health and vehicle and habitat factors cause changes to individual readiness. Those, along with team functionality, cause changes to crew capability. Additionally, the effects of system design and limitations impact the realistic chances of successfully performing a mission critical task. These are shown as vehicle and habitat factors, independent of effects on individual readiness or crew capability. For example, if the task is to repair a broken exercise device but spare parts for the device are not available because of mass constraints, the likelihood of successful task performance drops no matter the readiness of individuals or crew to perform the needed repairs. How the vehicle or habitat is designed can affect both individuals and an entire crew, for example the lack of individual quarters for sleep and privacy. However, vehicle and habitat factors can affect the likelihood of successful task performance, or in the case of mission critical tasks they can cause loss of mission objectives. If enough mission objectives are lost, then loss of mission may occur. Figure 3 is not an official HSRB DAG but is used here to communicate the concept. The point of this DAG is to ensure that SMEs consider multiple causes of decreased performance when thinking about risk, and how these effects on performance can lead to mission-level outcomes. Other nodes could be reasonably included in this DAG depending on their importance to the intended story. A graphical story such as this has been created for each of the 30 risks and the process is more thoroughly documented elsewhere^19^. Each of the DAGs are formally managed and tracked by the HSRB as part of the continuous risk management process^15^. The benefit of these diagrams primarily lies in communication, but they can help identify how and where specific factors contributing to a human system risk affect the larger system. In this sense, these diagrams are used to help highlight where gaps in knowledge or capability exist.

## Outlook and summary

This paper illustrates the evolution of the human system risk management process in recent years. These updates build on the formalized processes that are already in place. Updates to the risk scoring mechanisms and matrix ensure that the risk matrix more clearly conveys the current risk posture. Detail was added to the definitions, processes, and principles that the HSRB uses to provide guidance on how to approach human system risk management. Although the current updates were implemented in response to recognized limitations of prior approaches, they are not likely to solve all the challenges faced in this arena. It is fully expected that after several years of implementing these updates, further revisions will be needed. The process of continuous risk management used by the HSRB must continue to evolve in the face of changing needs of NASA and of the larger spaceflight industry. Extrapolation of these processes to commercial entities would likely require significant discussion of the driving goals of each business and whether the level of detail involved in this process would be useful outside of NASA. However, NASA concepts and approaches may be valuable for other health and science agencies to review as they make updates and evolve their human health risk assessment processes and procedures. The authors hope that this update will continue to raise public awareness of the current approaches used for managing human system risk at NASA and stimulate discussion about how to improve these processes for future space missions.

### Reporting summary

Further information on research design is available in the Nature Research Reporting Summary linked to this article.

### Supplementary information

Reporting Summary

## Data Availability

Data sharing is not applicable to this article as no datasets were generated or analyzed during the current study. The Human System Risk Board at NASA maintains a public website where further information and documentation can be found: https://www.nasa.gov/hhp/hsrb. The governing document for risk management JSC-66705 can be found at the NASA Technical Reports Server at https://ntrs.nasa.gov/citations/20205008887. Formal guidance on the NASA DAGs is publicly available at https://ntrs.nasa.gov/citations/20220006812. The HSRB approved DAGs for each Human System Risk are publicly available at https://ntrs.nasa.gov/citations/20220015709.
